# Association between plant-based dietary pattern and biological aging trajectory in a large prospective cohort

**DOI:** 10.1186/s12916-023-02974-9

**Published:** 2023-08-17

**Authors:** Sicong Wang, Wenyuan Li, Shu Li, Huakang Tu, Junlin Jia, Wenting Zhao, Andi Xu, Wenxin Xu, Min Kuang Tsai, David Ta-Wei Chu, Chi Pang Wen, Xifeng Wu

**Affiliations:** 1 Department of Big Data in Health Science School of Public Health, and Center of Clinical Big Data and Analytics of The Second Affiliated Hospital, Zhejiang University School of Medicine, Hangzhou, Zhejiang China; 2 Alibaba-Zhejiang University Joint Research Center of Future Digital Healthcare, Hangzhou, Zhejiang China; 3 Institute of Population Health Sciences, National Health Research Institutes, Zhunan, Taiwan; 4 MJ Health Management Center, Taipei, Taiwan; 5 Graduate Institute of Biomedical Sciences, College of Medicine, China Medical University, Taichung, Taiwan; 6 Department of Medical Research, China Medical University Hospital, Taichung, Taiwan; 7 National Institute for Data Science in Health and Medicine, Zhejiang University, Hangzhou, Zhejiang China; 8 Cancer Center, Zhejiang University, Hangzhou, Zhejiang China

**Keywords:** Aging trajectory, All-cause mortality, Plant-based diet index, Diet quality, Cohort

## Abstract

**Background:**

Aging is a dynamic and heterogeneous process that may better be captured by trajectories of aging biomarkers. Biological age has been advocated as a better biomarker of aging than chronological age, and plant-based dietary patterns have been found to be linked to aging. However, the associations of biological age trajectories with mortality and plant-based dietary patterns remained unclear.

**Methods:**

Using group-based trajectory modeling approach, we identified distinctive aging trajectory groups among 12,784 participants based on a recently developed biological aging measure acquired at four-time points within an 8-year period. We then examined associations between aging trajectories and quintiles of plant-based dietary patterns assessed by overall plant-based diet index (PDI), healthful PDI (hPDI), and unhealthful PDI (uPDI) among 10,191 participants who had complete data on dietary intake, using multivariable multinomial logistics regression adjusting for sociodemographic and lifestyles factors. Cox proportional hazards regression models were applied to investigate the association between aging trajectories and all-cause mortality.

**Results:**

We identified three latent classes of accelerated aging trajectories: slow aging, medium-degree, and high-degree accelerated aging trajectories. Participants who had higher PDI or hPDI had lower odds of being in medium-degree (OR = 0.75, 95% CI: 0.65, 0.86 for PDI; OR = 0.73, 95% CI: 0.62, 0.85 for hPDI) or high-degree (OR = 0.63, 95% CI: 0.46, 0.86 for PDI; OR = 0.62, 95% CI: 0.44, 0.88 for hPDI) accelerated aging trajectories. Participants in the highest quintile of uPDI were more likely to be in medium-degree (OR = 1.72, 95% CI: 1.48, 1.99) or high-degree (OR = 1.70, 95% CI: 1.21, 2.38) accelerated aging trajectories. With a mean follow-up time of 8.40 years and 803 (6.28%) participants died by the end of follow-up, we found that participants in medium-degree (HR = 1.56, 95% CI: 1.29, 1.89) or high-degree (HR = 3.72, 95% CI: 2.73, 5.08) accelerated aging trajectory groups had higher risks of death than those in the slow aging trajectory.

**Conclusions:**

We identified three distinctive aging trajectories in a large Asian cohort and found that adopting a plant-based dietary pattern, especially when rich in healthful plant foods, was associated with substantially lowered pace of aging.

**Supplementary Information:**

The online version contains supplementary material available at 10.1186/s12916-023-02974-9.

## Background

The number of individuals aged 65 years and older is increasing at an unprecedented pace, and it is expected to reach 1.6 billion by 2050 [1]. Human life expectancy has significantly increased in the past decades, potentially through advances in public health and healthcare services [2, 3]. However, population aging has been associated with increased risks of chronic diseases, resulting from physical or cognitive disability and mortality. Aging and age-related diseases present great challenges to current medical care, economy, and society as a whole and have become a global public health priority [4]. There is a critical need to identify modifiable risk factors that could help preserve overall health and good quality of life during aging.

Since aging is a dynamic and heterogeneous process of complex biological system, it may better be captured by repeated measures of aging biomarkers along the years. Recently, biological age has been advocated as a more accurate indicator of aging than chronological age, which integrates multiple objective biomarkers relevant to physical functions and provides information about overall health status [5], and it has been linked to both aging-related morbidity and mortality [6, 7]. It is therefore reasonable to believe that trajectories of biological age are associated with mortality, and identifying biological age trajectories that are linked to higher mortality will provide valuable insights and supportive evidence for early intervention and risk stratification. However, few studies have examined the longitudinal pattern of biological age as well as its associations with mortality.

Nutrition and diet have been advocated as major determinants of health [8]. Particularly, plant-based foods have been demonstrated as one of the major modifiable lifestyle factors for the prevention of age-related diseases and the preservation of overall good health status [9–11]. Accumulating evidence from epidemiological studies has shown that a plant-based dietary pattern is associated with decreased risk of mortality [12–15] and improved age-related neurological health including lower risks of dementia [16] and cognitive impairment [17]. Several studies have reported that plant-based foods were positively associated with successful or healthy aging [18–21]. For example, adherence to the “fruit” pattern was positively associated with successful aging which were absence of major chronic diseases while maintaining good mental health [18], but limited evidence is available with respect to an overall plant-based dietary recommendations. The studies on plant-based diet and aging generally measured aging by the presence of selected diseases or health status at one time point [18–21]. The time course of one’s aging process with respect to plant-based diet is less known. Consequently, evidence on the relationship between plant-based dietary patterns and aging trajectory is limited, especially in an Asian population.

Therefore, in the large prospective MJ cohort, we first established aging trajectories based on a newly developed biological aging index and assessed the associations between the aging trajectories with all-cause mortality; then, we evaluated the impacts of plant-based dietary patterns on these aging trajectories.

## Methods

### Study population

The study population was obtained from a standard medical screening program with the same models of instruments conducted by the MJ Health Management Institution. Recruitment happened at four geographically representative clinics located in the northern, northwest, central, and southern parts of Taiwan. Detailed information on the study design, methods, and rationale of the MJ prospective cohort has been reported previously [22–24]. Upon enrollment, every participant completed a self-administered questionnaire on demographic information, lifestyle, and medical history and underwent a series of anthropometric measurements, physical examinations, and blood and urinary tests at the time of the health check-ups.

In order to establish aging trajectories, we included 12,784 participants who were enrolled between 1996 and 2011, aged 50 years or older, and had completed data on the multi-dimensional aging measure (MDAge) at four times within an 8-year period after initial enrollment. To examine the associations between plant-based dietary patterns and aging trajectories, we further excluded 2593 (20.34%) participants who had incomplete data on food intake, leaving 10,191 participants for the analysis.

### Data collection

Data for sociodemographic characteristics (age, sex, education, and marital status), lifestyle factors (smoking status, alcohol consumption), food intake frequency, and personal and family medical history (e.g., stroke, cancer, and COPD) were collected by a self-administered questionnaire at baseline, and trained staffs provided detailed information if needed. Physical examinations were conducted by trained staffs with calibrated instruments following standard protocols, and height, weight, blood pressure, and lung function were measured. A 10-mL fasting blood sample from each participant was collected for laboratory tests at every physical examination. A timeline of data collection was shown in Additional file 1: Fig. S1.

Education (middle school or below, high school, junior college, college or above), marital status (unmarried, married), smoking status (never, ever), and drinking status (never, ever) were modeled as categorical variables. Body mass index (BMI) was calculated by dividing weight in kilograms by height in meters squared. Diabetes status was defined as having fasting blood glucose ≥ 126 mg/dL, having been diagnosed with diabetes by a doctor, or reported receiving anti-diabetic medication treatment. Hypertension was similarly defined as having systolic pressure ≥ 140 mm Hg, having been diagnosed with hypertension, or reported receiving anti-hypertensive medication treatment.

### Aging trajectory assessment

We calculated multi-dimensional aging measure (MDAge) in accordance with the method described previously [6]. Briefly, MDAge was calculated based on a linear combination of chronological age and 13 clinical chemistry biomarkers (lactate dehydrogenase, alkaline phosphatase, platelet count, forced expiratory volume in 1 s (FEV1), creatinine, systolic blood pressure, fasting blood glucose, BMI, gamma-glutamyl transpeptidase, albumin, leukocyte count, glutamic oxaloacetic transaminase and urea nitrogen) that were selected using random forest. The 14 biomarkers were then combined in a MDAge estimate using the Levine method. We then estimated MDAge acceleration (MDAgeAccel), calculated as the residuals resulting from a linear model regressing MDAge on chronological age. Therefore, MDAgeAccel represents MDAge after accounting for chronological age. A positive MDAgeAccel value indicates that the person is physiologically older than expected while a negative value refers to being younger than expected, based on one’s chronological age. Both MDAge and MDAgeAccel were calculated at four time points.

Distinct aging trajectories were then identified by group-based trajectory modeling (GBTM) approach based on MDAgeAccel at the four time points. The GBTM approach assumes that study population is comprised of a mixture of distinct groups defined by individual developmental trajectories [25]. The procedure was implemented in SAS PROC TRAJ [26]. Model selection is the critical procedure for GBTM analysis, and we followed suggestions from early studies to complete the task [27–30]. We first fit models with different numbers (from one to four) of trajectory groups and identified the optimal number of trajectory groups based on the log Bayes factor (2log_e_(B_10_)), calculated as 2(△BIC). This factor can be interpreted as the strength of evidence favoring the alternative model. Then, we determined the trajectory shapes that best describe the observed trajectories, and linear, quadratic, and cubic functions were all tried. The best-fitting trajectory models were selected based on the log Bayes factor, appropriate average posterior probability (> 0.7), and a minimum sample size in each group that accounted for > 5.0% of the overall population. More details of the GBTM approach have been described previously [26–35]. These distinct aging trajectories were comprised of individuals with similar aging acceleration patterns, displaying the dynamic and heterogeneous processes of aging.

### Dietary assessment

We evaluated the plant-based dietary patterns by constructing the plant-based diet index (PDI), healthful plant-based diet index (hPDI), and unhealthful plant-based diet index (uPDI), following an adapted approach used by Satija et al. [36].

Self-reported information on dietary intake was collected at first examination using a standardized and validated semi-quantitative food frequency questionnaire (FFQ) [37, 38]. This simplified FFQ had a total of 22 questions that asked about the frequency of intake of certain amount for 22 food items/groups at baseline. A total of 14 questions were asked corresponding to 14 food groups and were used to assess the plant-based dietary patterns in the current study. These 14 food groups were then grouped into plant foods and animal foods: the plant foods included whole grains, fresh vegetables, fresh fruit, rhizomes, legume, sugar, refined grains, and salt-preserved vegetable, and the animal foods included milk, dairy products, fish and aquatic products, eggs, meat, and pluck. Participants were asked how often on average during the previous year they had consumed each food of a standard portion size. We first defined intake frequency scores for each food item. For most food groups, including whole grains, rhizomes, legume, sugar, refined grains, salt-preserved vegetable, milk, dairy products, fish and aquatic products, eggs, meat, and pluck, the intake frequency “ > 1 serving/day,” “1 serving/day,” “4–6 servings/week,” “1–3 servings/week,” or “rarely or never” were assigned scores of 5, 4, 3, 2, and 1, respectively. For fruit and vegetables, average serving sizes per day were calculated firstly and then grouped into 5 categories after ranking from high to low and scored 5, 4, 3, 2, and 1, respectively. The 14 food group scores for each individual were summed to obtain the three indices, with a theoretical range of 14 to 70.

To calculate PDI, we assigned scores for each food group according to the intake frequency: a score of 5 was assigned for the highest frequency and 1 for the lowest frequency regarding the intake of plant food (positive scores); a score of 1 was assigned for the highest frequency and 5 for the lowest frequency regarding the intake of animal food (reverse scores). Therefore, the higher the frequency of eating plant foods and the lower the frequency of eating animal food, the higher the PDI.

However, not all plant-based foods are beneficial to aging in the long term. The healthy PDI (hPDI) and unhealthy PDI (uPDI) were created to represent healthy and unhealthy versions of a plant-based diet. Based on the most recent evidence [12, 36], we further classified the abovementioned plant foods into two categories: healthy plant foods (whole grains, fruits, vegetables, rhizomes, legumes) and less healthy plant foods (sugar, refined grains, and salt-preserved vegetable). To calculate hPDI, we assigned positive scores to healthy plant food groups and reverse scores to less healthy plant and animal food groups. To calculate uPDI, we assigned positive scores to less healthy plant food groups and reverse scores to healthy plant and animal food groups. In this way, hPDI and uPDI accounted for the quality of plant foods in calculating PDI. More details on constructing PDI, hPDI, and uPDI can be found in Additional file 1: Table S1. Considering that animal food group might deviate from the dimension as for the healthy or unhealthy plant foods, we reconstructed hPDI and uPDI by excluding animal food scores.

### Mortality and morbidity ascertainment

We calculated the follow-up time from the date of the fourth physical examination to the date of death or December 31, 2011, whichever came first. Mortality data were obtained by linking study participants to the Taiwan death file through 2011 using the unique identification numbers assigned to the residents.

Data on chronic disease conditions including stroke, cancer, COPD, osteoarthritis, diabetes mellitus, hypertension, and myocardial infarction were collected by self-reported questionnaires at the first physical examination. A chronic disease count variable ranging from 0 to 7 was created with 4 categories: 0, 1, 2, and 3 or more.

### Statistical analysis

The baseline characteristics of participants at the first physical examination were summarized using descriptive statistics. Spearman correlation analysis was conducted for food groups and dietary indices.

We applied multivariable multinomial logistics regression to estimate the associations between quintiles of PDI, hPDI, or uPDI and aging trajectories, with the lowest quintile as the reference for the exposure and slow aging as the reference category for the outcome. Odds ratios (ORs) and 95% confidence intervals (CIs) were estimated. Trend tests were conducted by assigning the median value to each quintile of an index and entering as a continuous variable in the model. In addition, we treated these indexes as continuous variables (per 10-unit increment) in our analyses. We also conducted sex- and age-stratified analyses to evaluate the associations between PDI, hPDI, or uPDI and aging trajectories. We used multinomial logistics regression models adjusting for age, gender, education level, marital status, smoking status, drinking status, and disease count at baseline. ORs and 95% CIs were reported. Moreover, we investigated the associations of 14 individual food items with aging trajectories using multinomial logistics regression models adjusting for age and gender.

Cox proportional hazards models were used to estimate the hazard ratios (HRs) and 95% CIs for the associations between aging trajectories and all-cause mortality. For this analysis, the follow-up started at the fourth physical examination. A three-stage modeling process was adopted. Model 1 adjusted for age, gender, and MDAgeAccel (continuous) at baseline. Model 2 additionally adjusted for education level, marital status, smoking status, and drinking status. Model 3 further adjusted for disease count at baseline. We then used Kaplan–Meier survival curves and log-rank tests to estimate the incidence of mortality by aging trajectories in all participants and gender-stratified groups. We conducted complete case analyses, and participants with missing covariates data were excluded from corresponding analysis.

All analyses were performed using SAS 9.3 (SAS Institute, Cary, NC), and plots were created using R 3.5.3. A two-sided *p*-value < 0.05 was considered statistically significant.

## Results

### Sample characteristics

Table 1 presents the baseline characteristics of the 12,784 enrolled participants. Their mean (± SD) age was 58.60 ± 6.51 years and 52.61% were women. About 73.48% and 73.31% were never smokers and never drinkers, and 47.70% of the participants were free of chronic disease at baseline. The mean duration of the follow-up was 8.40 years (SD = 3.58), and 803 participants died by the end of follow-up. The pairwise Spearman correlation coefficients between different 14 food groups and 3 constructed food indices were shown in Additional file 1: Fig. S2.

**Table 1:** Baseline characteristics of all participants

**Characteristics**	**Aging trajectory**			**Total** 12,784 (100%)
	**Slow aging** 6031 (47.18%)	**Medium-degree** 6090 (47.63%)	**High-degree** 663 (5.19%)	
Age, years^a^	58.00 (6.43)	59.08 (6.52)	59.61 (6.67)	58.60 (6.51)
MDAgeAccel, years^a^	− 1.32 (2.28)	2.46 (2.46)	8.34 (4.56)	0.98 (3.57)
Gender				
Male	2873 (47.64)	2880 (47.29)	305 (46.00)	6058 (47.39)
Female	3158 (52.36)	3210 (52.71)	358 (54.00)	6726 (52.61)
Education				
Middle school or below	3200 (54.64)	4008 (67.93)	489 (76.77)	7697 (62.11)
High school	1111 (18.97)	912 (15.46)	78 (12.24)	2101 (16.95)
Junior college	624 (10.66)	455 (7.71)	36 (5.65)	1115 (9.00)
College or above	921 (15.73)	525 (8.90)	34 (5.34)	1480 (11.94)
Marital status				
Unmarried	880 (15.21)	877 (14.97)	124 (19.81)	1881 (15.33)
Married	4906 (84.79)	4982 (85.03)	502 (80.19)	10,390 (84.67)
Smoking				
Never	4254 (76.10)	3910 (71.64)	393 (65.83)	8557 (73.48)
Ever	1336 (23.90)	1548 (28.36)	204 (34.17)	3088 (26.52)
Drinking				
Never	4099 (74.26)	3993 (72.68)	417 (70.32)	8509 (73.31)
Ever	1421 (25.74)	1501 (27.32)	176 (29.68)	3098 (26.69)
Disease count				
0	3623 (60.07)	2370 (38.92)	105 (15.84)	6098 (47.70)
1	1965 (32.58)	2741 (45.01)	301 (45.40)	5007 (39.17)
2	389 (6.45)	836 (13.73)	220 (33.18)	1445 (11.30)
3 or more	54 (0.90)	143 (2.34)	37 (5.58)	234 (1.83)
Death	187 (3.10)	469 (7.70)	147 (22.17)	803 (6.28)
PDI^a^	40.93 (3.59)	40.42 (3.72)	40.30 (3.66)	40.66 (3.66)
hPDI^a^	50.21 (3.67)	49.78 (3.79)	49.70 (3.97)	49.99 (3.75)
uPDI^a^	46.80 (4.47)	47.66 (4.49)	47.60 (4.75)	47.24 (4.51)

Slow aging: slow aging trajectory, Medium-degree: medium-degree accelerated aging trajectory, High-degree: high-degree accelerated aging trajectory

Data were presented as No. (%). There were missing data on education (*n* = 391), marital status (*n* = 513), smoking status (*n* = 1139), drinking status (*n* = 1177), PDI or hPDI or uPDI (*n* = 2593). ^a^Mean (SD) was reported

### Aging trajectories and all-cause mortality

**Fig. 1:**
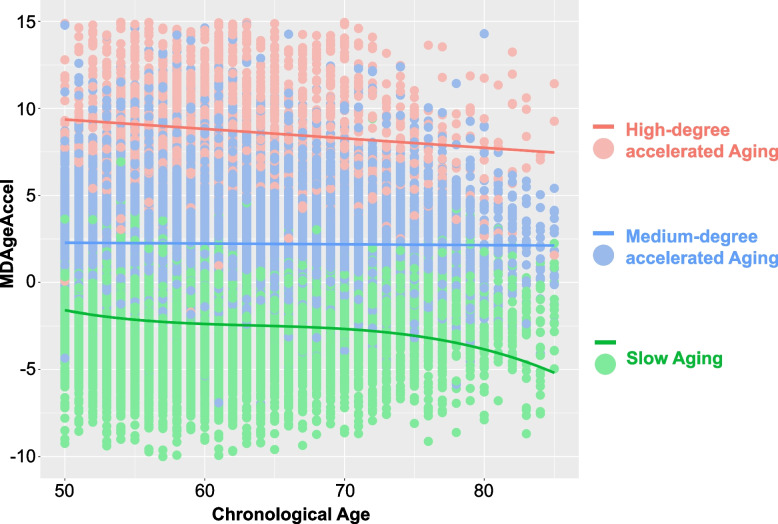
Aging trajectories in MJ cohort. Three trajectory classes identified: Slow aging, medium-degree accelerated aging trajectory, and high-degree accelerated aging trajectory. The different colors of points represent raw data points of participants in different aging trajectories. The *Y*-axis represents aging acceleration, and x-axis is for chronological age

Three latent classes of accelerated aging trajectories were identified by GBTM and were illustrated in Fig. 1. The model selection criteria of trajectory groups were shown in Additional file 1: Table S2. About 47.18% of the study participants were grouped in the slow aging trajectory, all had negative MDAgeAccel values, and these individuals had generally smaller biological age than their chronological age over the four measurements. About 47.63% of the study participants were grouped in the medium-degree accelerated aging trajectory, and these individuals had displayed a medium level of aging acceleration in the beginning, along with almost no change throughout the whole period. Individuals in the medium-degree accelerated aging group had moderate morbidity (61.08%) at baseline and moderate mortality (7.70%) during the follow-up time (Table 1). The rest of the study participants (5.19%) were in the high-degree accelerated aging group, displaying a consistently high level of accelerated aging over the four measurements. Moreover, 22.17% of the individuals in the high-degree accelerated aging group died during the follow-up time (Table 1). In general, the trajectories are homogeneous across key subgroups (Additional file 1: Fig. S3).

**Fig. 2:**
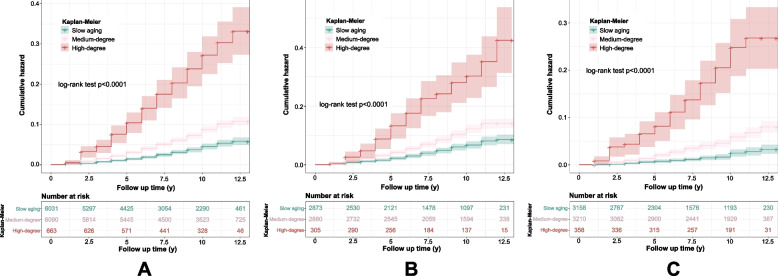
Kaplan–Meier estimates of the cumulative incidence of mortality by aging trajectory groups. **a** Total participants, **b** male, and **c** female. Slow aging trajectory, Medium-degree: medium-degree accelerated aging trajectory, High-degree: high-degree accelerated aging trajectory

**Fig. 3:**
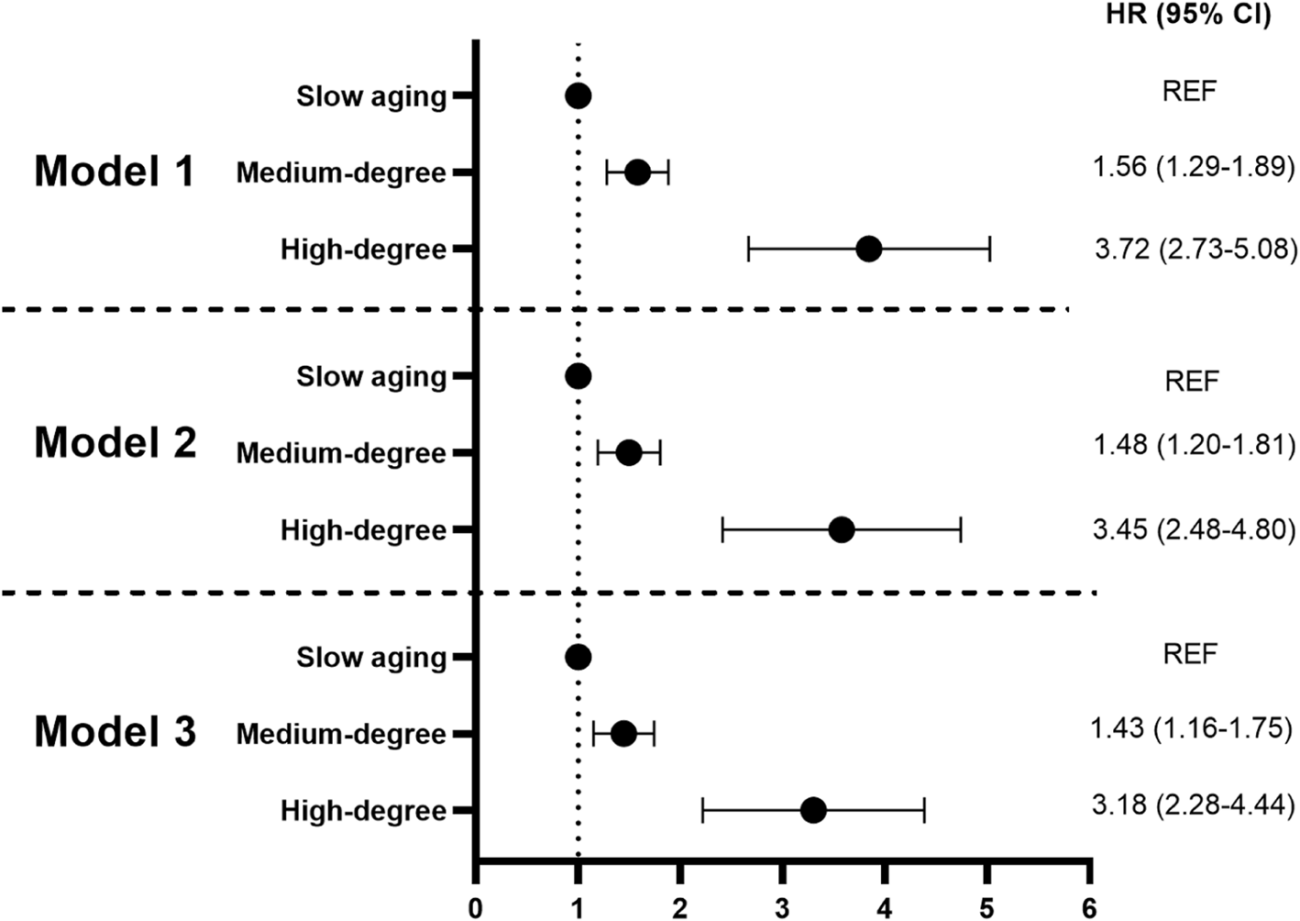
Multivariable-adjusted HRs and 95% CIs of all-cause mortality according to aging trajectories. Slow aging trajectory was as a reference group. Slow aging: slow aging trajectory. Medium-degree: medium-degree accelerated aging trajectory. High-degree: high-degree accelerated aging trajectory. Model 1 adjusted for MDAgeAccel at baseline, age, and gender; model 2 additionally adjusted for education level, marital status, smoking status, and drinking status; model 3 further adjusted for disease count based on model 2

Based on the Kaplan–Meier curve, differential risks of mortality were observed for each aging trajectory (Fig. 2A), and the association pattern was consistently seen in both sexes (Fig. 2B, C). Figure 3 illustrated the results from Cox proportional-hazards models. Participants in medium-degree accelerated aging trajectory had 1.56-fold (HR = 1.56, 95% CI: 1.29, 1.89) higher risk and those in high-degree accelerated aging trajectory had 3.72-fold (HR = 3.72, 95% CI: 2.73, 5.08) higher risk of all-cause mortality than participants in the slow aging trajectory over the follow-up. The associations attenuated slightly after additionally adjusting for education, marital status, and health behaviors.

### Overall plant-based dietary patterns and long-term aging trajectory

PDI score was slightly higher among the participants in the slow aging trajectory (Table 1). Table 2 shows the results of multivariate multinomial logistic regression models examining the relations between plant-based dietary patterns and accelerated aging trajectories. Participants in the highest quintile of PDI had 25% lower odds of being in the medium-degree accelerated aging trajectory (OR = 0.75, 95% CI: 0.65, 0.86) and 37% lower odds of being in the high-degree accelerated aging trajectory (OR = 0.63, 95% CI: 0.46, 0.86) compared to participants in the lowest quintile. Linear regression analysis showed that a 10-unit increment in PDI was associated with lower odds of being in the medium-degree accelerated trajectory (OR = 0.74, 95% CI: 0.66, 0.84) or lower odds of being in the high-degree accelerated trajectory (OR = 0.70, 95% CI: 0.53, 0.92).

**Table 2:** Associations between quintiles of PDI, hPDI, and uPDI and aging trajectories based on multinomial logistic regression model

Diet index	Median of diet index	Medium-degree vs slow aging			High-degree vs slow aging		
		*N*^a^	OR (95% CI)	*p*-trend	*N*^a^	OR (95% CI)	*p*-trend
**Quintile of PDI (*N*)**							
Q1 (2402)	36	1241	Ref	< 0.0001	145	Ref	0.0050
Q2 (2038)	39	969	0.89 (0.77, 1.03)		96	0.67 (0.49, 0.92)	
Q3 (2072)	40	938	0.78 (0.68, 0.90)		93	0.67 (0.50, 0.91)	
Q4 (1753)	42	723	0.69 (0.59, 0.79)		71	0.62 (0.45, 0.86)	
Q5 (1926)	45	822	0.75 (0.65, 0.86)		89	0.63 (0.46, 0.86)	
**Per 10, unit increment of PDI**			0.74 (0.66, 0.84)			0.70 (0.53, 0.92)	
**Quintile of hPDI (*N*)**							
Q1 (2080)	45	1053	Ref	< 0.0001	118	Ref	0.0004
Q2 (1787)	48	869	0.88 (0.77, 1.01)		92	0.79 (0.59, 1.06)	
Q3 (2144)	49	956	0.76 (0.64, 0.90)		99	0.62 (0.42, 0.91)	
Q4 (1981)	51	874	0.71 (0.62, 0.81)		85	0.58 (0.43, 0.78)	
Q5 (2199)	54	941	0.73 (0.62, 0.85)		100	0.62 (0.44, 0.88)	
**Per 10, unit increment of hPDI**			0.73 (0.65, 0.82)			0.65 (0.50, 0.85)	
**Quintile of uPDI (*N*)**							
Q1 (1851)	41	733	Ref	< 0.0001	78	Ref	0.0114
Q2 (2059)	45	888	1.11 (0.97, 1.28)		109	1.27 (0.92, 1.74)	
Q3 (1809)	47	824	1.18 (1.02, 1.36)		72	0.93 (0.65, 1.31)	
Q4 (2316)	50	1095	1.27 (1.11, 1.45)		114	1.24 (0.90, 1.70)	
Q5 (2156)	53	1153	1.72 (1.48, 1.99)		121	1.70 (1.21, 2.38)	
**Per 10, unit increment of uPDI**			1.43 (1.30, 1.58)			1.39 (1.11, 1.75)	

Slow aging: slow aging trajectory, Medium-degree: medium-degree accelerated aging trajectory, High-degree: high-degree accelerated aging trajectory. *N*^a^: Number of participants in medium-degree accelerated aging trajectory or high-degree accelerated aging trajectory. Model adjusted for age, gender, education level, marital status, smoking status, drinking status, and chronic disease count

### Associations of hPDI and uPDI with long-term aging trajectory

The hPDI score was slightly higher while uPDI score was lower among the participants in the slow aging trajectory, and the details were shown in Table 1.

The results of the associations of hPDI and uPDI with accelerated aging trajectories were shown in Table 2. Those in the highest quintile of hPDI had 27% lower odds of belonging to the medium-degree accelerated aging trajectory (OR = 0.73, 95% CI: 0.62, 0.85) and 38% lower odds of being in the high-degree accelerated aging trajectory (OR = 0.62, 95% CI: 0.44, 0.88) (Table 2). In contrast, the highest quintile of uPDI was associated with 72% higher odds of belonging to medium-degree accelerated aging trajectory (OR = 1.72, 95% CI: 1.48, 1.99) and 70% higher odds of being in high-degree accelerated aging trajectory (OR = 1.70, 95% CI: 1.21, 2.38) (Table 2).

Linear regression analysis indicated that a 10-unit increment in hPDI was associated with lower odds of being in the medium-degree accelerated trajectory (OR = 0.73, 95% CI: 0.65, 0.82) or of being in the high-degree accelerated trajectory (OR = 0.65, 95% CI: 0.50, 0.85). Whereas a 10-unit increment in uPDI was associated with 43% higher odds of being in the medium-degree accelerated trajectory (OR = 1.43, 95% CI: 1.30, 1.58) or 39% higher odds of being in the high-degree accelerated trajectory (OR = 1.39, 95% CI: 1.11, 1.75). Detailed results were listed in Table 2.

We generated reconstructed hPDI (rhPDI) and reconstructed uPDI (ruPDI) by excluding animal food scores and investigated the associations of rhPDI and ruPDI with the trajectory groups. The correlation matrix among plant-based food groups and reconstructed food indices was provided in Additional file 1: Figure S4. We found that those in the highest quintile of rhPDI still had 46% lower odds of being to the medium-degree accelerated aging trajectory (OR = 0.54, 95% CI: 0.47, 0.63) and 50% lower odds of being in the high-degree accelerated aging trajectory (OR = 0.50, 95% CI: 0.36, 0.68) (Additional file 1: Table S3). In contrast, the highest ruPDI was associated with 86% higher odds of being to medium-degree accelerated aging trajectory (OR = 1.86, 95% CI: 1.60, 2.15) and a 101% higher odds of being in high-degree accelerated aging trajectory (OR = 2.01, 95% CI: 1.46, 2.76) (Additional file 1: Table S3).

We further conducted sex- and age-stratified analyses (Table 3). Overall, the associations were consistent with our primary findings: adopting an overall (higher PDI) and healthy plant-based dietary pattern (higher hPDI) were associated with substantially lower odds of being in higher accelerated aging trajectories, and adopting an unhealthful plant-based dietary pattern (higher uPDI score) was associated with substantially higher odds of being in higher accelerated aging trajectories.

**Table 3:** Associations between quintiles of PDI, hPDI and uPDI and aging trajectories based on multinomial logistic regression model in age- and sex-stratified groups

Diet index	Medium-degree vs slow aging (OR, 95% CI)						High-degree vs slow aging (OR, 95% CI)					
	Q1	Q2	Q3	Q4	Q5	*p*-trend	Q1	Q2	Q3	Q4	Q5	*p*-trend
**PDI**												
Male	Ref	0.91 (0.74, 1.11)	0.76 (0.63, 0.93)	0.78 (0.64, 0.95)	0.82 (0.67, 1.00)	0.0186	Ref	0.94 (0.60, 1.46)	0.77 (0.50, 1.20)	0.89 (0.56, 1.40)	0.79 (0.50, 1.25)	0.3145
Female	Ref	0.86 (0.70, 1.05)	0.79 (0.65, 0.96)	0.60 (0.49, 0.74)	0.69 (0.56, 0.84)	< 0.0001	Ref	0.47 (0.30, 0.74)	0.56 (0.37, 0.86)	0.43 (0.27, 0.68)	0.50 (0.32, 0.76)	0.0030
Age < 60	Ref	0.85 (0.71, 1.03)	0.76 (0.64, 0.91)	0.68 (0.57, 0.82)	0.77 (0.64, 0.92)	0.0005	Ref	0.61 (0.40, 0.92)	0.52 (0.35, 0.77)	0.51 (0.33, 0.78)	0.53 (0.35, 0.80)	0.0018
Age ≥ 60	Ref	0.95 (0.76, 1.20)	0.82 (0.66, 1.03)	0.71 (0.56, 0.89)	0.74 (0.59, 0.93)	0.0005	Ref	0.84 (0.51, 1.40)	0.97 (0.60, 1.55)	0.83 (0.51, 1.37)	0.81 (0.50, 1.33)	0.4488
**hPDI**												
Male	Ref	0.85 (0.71, 1.02)	0.75 (0.59, 0.95)	0.69 (0.58, 0.84)	0.76 (0.61, 0.94)	0.0003	Ref	0.92 (0.61, 1.39)	0.83 (0.49, 1.41)	0.63 (0.41, 0.96)	0.73 (0.44, 1.21)	0.0335
Female	Ref	0.91 (0.74, 1.12)	0.76 (0.59, 0.97)	0.71 (0.58, 0.86)	0.70 (0.56, 0.88)	< 0.0001	Ref	0.64 (0.42, 0.99)	0.44 (0.25, 0.78)	0.50 (0.33, 0.77)	0.50 (0.31, 0.82)	0.0032
Age < 60	Ref	0.90 (0.76, 1.07)	0.77 (0.62, 0.96)	0.67 (0.56, 0.79)	0.73 (0.60, 0.89)	< 0.0001	Ref	0.81 (0.55, 1.19)	0.71 (0.44, 1.15)	0.48 (0.33, 0.72)	0.52 (0.32, 0.84)	0.0002
Age ≥ 60	Ref	0.88 (0.70, 1.10)	0.75 (0.57, 0.99)	0.78 (0.62, 0.98)	0.75 (0.58, 0.96)	0.0127	Ref	0.77 (0.48, 1.24)	0.49 (0.26, 0.95)	0.72 (0.45, 1.15)	0.74 (0.44, 1.25)	0.3039
**uPDI**												
Male	Ref	1.06 (0.87, 1.28)	1.34 (1.10, 1.64)	1.23 (1.01, 1.49)	1.68 (1.35, 2.09)	< 0.0001	Ref	0.98 (0.64, 1.50)	0.98 (0.61, 1.57)	0.97 (0.63, 1.51)	1.51 (0.93, 2.43)	0.2175
Female	Ref	1.18 (0.97, 1.43)	1.04 (0.85, 1.28)	1.31 (1.08, 1.58)	1.75 (1.42, 2.15)	< 0.0001	Ref	1.73 (1.07, 2.78)	0.87 (0.51, 1.50)	1.59 (0.99, 2.55)	1.97 (1.20, 3.21)	0.0316
Age < 60	Ref	1.20 (1.00, 1.43)	1.26 (1.05, 1.51)	1.41 (1.18, 1.67)	1.78 (1.47, 2.16)	< 0.0001	Ref	1.50 (0.99, 2.30)	1.12 (0.70, 1.79)	1.42 (0.92, 2.20)	2.04 (1.29, 3.24)	0.0151
Age ≥ 60	Ref	1.01 (0.81, 1.25)	1.09 (0.87, 1.37)	1.08 (0.86, 1.34)	1.63 (1.28, 2.06)	0.0002	Ref	1.01 (0.62, 1.63)	0.73 (0.43, 1.24)	1.06 (0.66, 1.69)	1.41 (0.86, 2.31)	0.2164

Slow aging: slow aging trajectory, Medium-degree: medium-degree accelerated aging trajectory, High-degree: high-degree accelerated aging trajectory

Model adjusted for age, gender, education level, marital status, smoking status, drinking status, and chronic disease count

### Individual food items and aging trajectory

In terms of each food group, higher intake frequency of fresh fruits (OR_≥2 servings/day vs <0.5 servings/day_ = 0.70; 95% CI, 0.60, 0.82), fresh vegetables (OR_>4 servings/day vs <2 servings/day_ = 0.48; 95% CI, 0.42, 0.54), and legumes (OR_>1 serving/day vs never_ = 0.56; 95% CI, 0.39, 0.80) were potentially the main contributors to the beneficial associations of PDI and hPDI with aging; and higher intake frequency of refined grain (OR_>1 serving/day vs never_ = 1.57; 95% CI, 1.25, 1.97), salt-preserved vegetable (OR _1 serving/day vs never_ = 1.61, 95% CI, 1.21, 2.16), dairy products (OR _1 serving/day vs never_ = 1.33, 95% CI, 1.14, 1.56), and pluck (OR _1 serving/day vs never_ = 1.77; 95% CI, 1.43, 2.18) were primary contributors to the detrimental association of uPDI with aging (Additional file 1: Table S4).

## Discussion

In this prospective cohort study, we identified three aging trajectories where participants in medium-degree or high-degree accelerated aging trajectory groups had higher risks of death than those in the slow aging trajectory. We then found that adopting an overall plant-based dietary pattern was associated with lower odds of being in medium-degree or high-degree accelerated aging trajectories. Our study demonstrated a differential impact of plant-based foods on accelerated aging trajectory, i.e., a healthful plant-based diet was more beneficial to aging than an unhealthful plant-based diet. Fresh fruits, fresh vegetables, and legumes were major contributors found in our healthful plant-based diet analysis, whereas refined grain, salt-preserved vegetable, dairy products, and pluck were major contributors from unhealthful plant-based diet analysis.

Using the MDAge, a newly developed biological age biomarker, we successfully established three distinct aging trajectories in our study sample. It also allowed us to evaluate the pace of aging over the years. This biological age is arguably a more accurate measure of aging than chronological age [6], because it integrates multiple biomarkers relevant to physical functions and provides information about overall health. MDAge (AUC = 0.891, 95% CI: 0.888, 0.894) outperformed the model with chronological age (AUC = 0.868, 95% CI: 0.865, 0.872) in predicting all-cause mortality [6]*.* Several earlier studies [39–45] have identified different patterns of aging trajectories in older adults by measuring frailty index [39], health and functioning items [40, 41], physical ability and mental health items [42, 43], or other subjective indicators [44, 45]. For example, a study identified three types of healthy aging trajectories (stable-good aging well, initially aging well then deteriorating, and stable-poor) from a prospective 16-year longitudinal study of 1000 older Australians, in which “aging well” was defined based on self-rated health, psychological wellbeing, and independence in daily living [45]. Compared to previous studies, our current study demonstrated the feasibility of estimating pace of aging over a long period of time in this Asian population. Better than measuring aging at a single time point, establishing aging trajectories based on aging biomarkers measured over a longer time period may capture the dynamic and heterogeneous nature of aging and, expectedly, identify precisely different population with varying risks of age-related diseases or mortality. Figure 1 showed a gradual decrease of the trajectory curves in both high-degree and slow aging trajectory groups after 80 years old. However, this decreasing trend was statistically insignificant. A possible explanation was that among older age groups, only those who were biologically younger could survive, which lowered the average level of MDAge and MDAgeAccel in these groups. Additional file 1: Fig. S3 also indicated potential heterogeneity in trajectories between different subgroups. However, the small number of participants in each trajectory at age 80 or above restricted us from further analyses.

Diet is one of the primary modifiable lifestyle factors, and plant-based dietary patterns prevent the development of chronic diseases [46–48] and promote better age-related neurological health, such as a lower risk of dementia [16] and cognitive impairment [17]. The plant-based dietary pattern is generally defined as a higher intake of various plant foods (such as fruits and vegetables, whole grains, and legume) and lower consumption of animal foods. Individual plant-based food group has been linked to slower biological aging [18–20]. Among 6308 older adults from the Melbourne Collaborative Cohort Study, adherence to the “fruit” pattern was positively associated with absence of major chronic diseases and major limitations of physical function while maintaining good mental health (i.e., successful aging), and adherence to the “meat/fatty” pattern was inversely associated with successful aging over a mean of 11.7-year of follow-up [18]. Quach et al. examined cross-sectional data from 4575 individuals and showed that the levels of blood biomarkers for fruit/vegetable consumption specifically were significantly correlated with slower epigenetic aging [19]. However, most of the published studies lacked comprehensive assessment of plant-based dietary patterns, limiting the strength of the evidence and the feasibility of converting their findings into intervention or policy making. In this study, we included 14 food groups (whole grains, fresh vegetables, fresh fruit, rhizomes, legume, sugar, refined grains, salt-preserved vegetable, milk, dairy products, fish and aquatic products, eggs, meat, and pluck) for the assessment plant-based dietary patterns. In addition, plant-based dietary indices were more optimal in several aspects. For example, they primarily focus on the source of food rather than prior knowledge of the relationship between food and health by negatively weighing all animal foods and positively weighing all plant foods.

Moreover, as not all plant-based foods are necessarily beneficial, we adapted hPDI and uPDI scores [12, 36] to represent healthful and unhealthful versions of a plant-based diet. The hPDI emphasized on plant-based foods known to be associated with improved health outcomes whereas uPDI emphasized on less healthy plant-based foods. Zhou et al. found that an overall and healthful plant-based dietary pattern was associated with 34% and 45% higher likelihood of healthy aging (defined as the absence of 10 chronic diseases by self-reported and the absence of age-related dysfunction), among individuals aged 45–74 years in Singapore [21]. While our analysis confirmed these previous findings, we further provided evidence regarding the associations between PDI and aging trajectories and demonstrated that both overall and healthy plant-based dietary patterns may de-accelerate one’s aging process. As plant-based dietary pattern is increasingly recommended for its contribution to environmental and source sustainability, its relation to aging has attracted a lot of attention. We observed that not all plant foods are necessarily beneficial to slow aging, and our findings supported the notion for recommending healthful, but not unhealthful, plant-based dietary patterns from a public health perspective. Furthermore, we investigated the associations of reconstructed hPDI and uPDI with aging trajectory. The results remained similar to the original findings.

We further showed that fresh fruits, fresh vegetables, and legumes were the primary drivers of this protective effect, which is aligned with previous studies. Higher intake of fruits and vegetables has been associated with lower mortality from all causes, cardiovascular disease and cancer [49], and with greater intrinsic capacity [50] or slower biological aging [51]. Along with fruits and vegetables, legumes also have been linked with healthy aging and longevity. A cross-cultural study conducted by International Union of Nutritional Sciences (IUNS) and the World Health Organization (WHO) revealed that for every 20 g increase in daily legumes intake, there was an 8% reduction in the risk of death (RR = 0.92, 95% CI 0.85, 0.99). The potential explanations for the health benefits may include the anti-inflammatory and antioxidant effects of dietary fibers and polyphenols [52–54].

Several epidemiological studies have reported the relationships between biological aging and well-known dietary patterns, such as Mediterranean diet [55–57], Dietary Approaches to Stop Hypertension (DASH) [55–57], Healthy Eating Index [55, 58], and Prudent dietary pattern [58]. In those studies, biological aging was measured by epigenetic aging clock [56], telomere length [55, 58], and blood biomarkers [57]. Their findings supported the hypothesis that high quality diet may slow aging. For example, Mediterranean diet, a dietary pattern that was associated with lower cardiovascular risk and longer survival [59, 60], was found to be inversely associated with epigenetic aging [57]. Mediterranean diet was characterized by high intake of plant foods, olive oil as the main source of added fat, moderate intake of fish and seafood, moderate consumption of poultry, dairy products and wine, and low consumption of red meat [61]. This dietary pattern is rich in plant-based foods, while not distinguishing the quality of the food items. Our study reported additional evidence regarding a significantly higher odds of accelerated aging associated with more frequent consumption of unhealthy plant-based foods. Quantitatively differentiated healthy plant-based foods versus unhealthy plant-based foods has significant impact on healthy aging in general. Furthermore, most of the published studies were cross-sectional studies in populations from Western countries. Prospective studies on dietary patterns and biological age at multiple time points are therefore in demand, especially in Asian populations.

A few limitations of the current study should be noted. First, our dietary assessment was based on a standardized and validated semi-quantitative food frequency questionnaire that was self-reported by the participants [37, 38], which may result in measurement errors. However, since dietary data was assessed before the assessment of aging trajectories, the measurement errors were likely non-differential, which may bias the findings towards null, and we still observed statistically significant associations between dietary patterns and aging trajectories. Second, plant-based dietary patterns were estimated primarily based on intake frequency. Given our food frequency questionnaire may not be able to comprehensively cover the food items/groups that were consumed in our study population, we may underestimate intakes of total energy and nutrients. Therefore, we did not estimate total energy intake or perform energy-adjustment. Nevertheless, the using food frequency questionnaire to assess dietary patterns have been demonstrated to be reliable and valid measures in several studies [36, 62–65]. Third, although we controlled for several lifestyle factors, the possibility of residual confounding cannot be excluded due to the observational nature of the study. Fourth, participants included in the current analysis were mainly middle-aged or elderly Asians, and both dietary patterns and MDAge were identified among this population. Therefore, our results may not be generalized to populations of other ethnicities.

There were also several strengths of the present study. First, we utilized a prospective design with a relatively large sample size and a long follow-up period. Second, instead of focusing on the presence of selected diseases or impairments, we conceptualized aging acceleration using multiple multi-dimensional indicators which were based on 14 routinely examined biomarkers representing multiple organ systems. Third, compared to a single time-point evaluation, we used the GBTM to trace aging acceleration at four-time points, which might be more appropriate in evaluating the aging process. The GBTM model accounts for the variations in time to distinguish changes in aging acceleration over time and is able to identify the heterogeneity of aging.

## Conclusions

In summary, we identified three distinct aging trajectories and showed that adopting healthy plant-based dietary patterns was associated with slow aging trajectory and that unhealthful plant-based diet led to an accelerated aging trajectory. Our findings suggested that increasing the intake of healthy plant-based foods while reducing the intake of unhealthy plant-based foods and certain animal foods slows down the aging process in an Asian population.

### Supplementary Information

**Additional file 1: Table S1.** Food groups and scoring. **Table S2.** Model selection criteria of trajectory subgroups. **Table S3.** Associations between quintiles of rhPDI and ruPDI and aging trajectories based on multinomial logistic regression model. **Table S4.** Odds ratios and 95% CIs for aging trajectories, according to individual food groups and categories variables frequency. **Fig. S1.** Timeline of data collection. **Fig. S2.** The pairwise Spearman correlation coefficient between different 14 food groups and 3 constructed food indices. **Fig. S3.** Aging trajectories across gender, education levels, married status, smoking status and drinking status. **Fig. S4.** The pairwise Spearman correlation coefficient between different plant-based food groups and constructed food indices (PDI and rhPDI and ruPDI).

## Data Availability

The datasets generated and/or analyzed during the current study are not publicly available. Data requests for original datasets can be submitted to the MJ Health Research Foundation and MJ Health Resource Center at http://www.mjhrf.org/. Please contact the corresponding authors for details.

## Abbreviations

BMI: Body mass index
CI: Confidence interval
FEV1: Forced expiratory volume in 1 s
GBTM: Group-based trajectory modeling
hPDI: Healthful plant-based diet index
HR: Hazard ratio
MDAge: Multi-dimensional aging measure
MDAgeAccel: MDAge acceleration
OR: Odds ratio
PDI: Plant-based diet index
uPDI: Unhealthful plant-based diet index
