# Supplementary material for: Association between plant-based dietary pattern and biological aging trajectory in a large prospective cohort
Source: BMC Med. 2023 Aug 17;21:310. doi: 10.1186/s12916-023-02974-9 (PMC10433678; doi:10.1186/s12916-023-02974-9)

**Association between plant-based dietary pattern and biological aging trajectory in a large prospective cohort**

**Additional file 1**

Sicong Wang, Wenyuan Li, Shu Li, Huakang Tu, Junlin Jia, Wenting Zhao, Andi Xu, Wenxin Xu, Min Kuang Tsai, David Ta-Wei Chu, Chi Pang Wen, Xifeng Wu

**Table of Contents**

**Table S1. Food groups and scoring………………………………………………………………….2**

**Table S2. Model selection criteria of trajectory subgroups………………………………...……...4**

**Table S3. Associations between quintiles of rhPDI and ruPDI and aging trajectories based on multinomial logistic regression model………………………………………………………………5**

**Table S4. Odds ratios and 95% CIs for aging trajectories, according to individual food groups and categories variables frequency……………….…………………………………………………6**

**Fig. S1. Timeline of data collection………………………………………………………………….8**

**Fig. S2. The pairwise Spearman correlation coefficient between different 14 food groups and**

**3 constructed food indices………………………………..………………………….………………9**

**Fig. S3. Aging trajectories across gender, education levels, married status, smoking status**

**and drinking status………………………………………………………………………..………...10**

**Fig. S4. The pairwise Spearman correlation coefficient between different plant-based food groups and constructed food indices (PDI and rhPDI and ruPDI) ………………………………………12**

**Table S1. Food groups and scoring**

| **Food category** | **Food** | **Frequency** | **PDI** | **hPDI** | **uPDI** |
| --- | --- | --- | --- | --- | --- |
| Plant food | Whole grain | >1 servings /day | 5 | 5 | 1 |
|  |  | 1 servings /day | 4 | 4 | 2 |
|  |  | 4-6 servings /week | 3 | 3 | 3 |
|  |  | 1-3 servings /week | 2 | 2 | 4 |
|  |  | Rarely or never | 1 | 1 | 5 |
|  | Fresh fruit | ≥ 2 servings/day | 5 | 5 | 1 |
|  |  | 1·5-2 servings/day | 4 | 4 | 2 |
|  |  | 1-1·5 servings/day | 3 | 3 | 3 |
|  |  | 0·5-1 servings/day | 2 | 2 | 4 |
|  |  | < 0·5 servings/day | 1 | 1 | 5 |
|  | Fresh vegetable | > 4 servings/day | 5 | 5 | 1 |
|  |  | 3-4 servings/day | 4 | 4 | 2 |
|  |  | 2·5-3 servings/day | 3 | 3 | 3 |
|  |  | 2-2·5 servings/day | 2 | 2 | 4 |
|  |  | < 2 servings/day | 1 | 1 | 5 |
|  | Rhizomes | >1 serving/day | 5 | 5 | 1 |
|  |  | 1 serving/day | 4 | 4 | 2 |
|  |  | 4-6 servings/week | 3 | 3 | 3 |
|  |  | 1-3 servings/week | 2 | 2 | 4 |
|  |  | Rarely or never | 1 | 1 | 5 |
|  | Legume | >1 serving/day | 5 | 5 | 1 |
|  |  | 1 serving/day | 4 | 4 | 2 |
|  |  | 4-6 servings/week | 3 | 3 | 3 |
|  |  | 1-3 servings/week | 2 | 2 | 4 |
|  |  | Rarely or never | 1 | 1 | 5 |
|  | Sugar | >1 serving/day | 5 | 1 | 5 |
|  |  | 1 serving/day | 4 | 2 | 4 |
|  |  | 4-6 servings/week | 3 | 3 | 3 |
|  |  | 1-3 servings/week | 2 | 4 | 2 |
|  |  | Rarely or never | 1 | 5 | 1 |
|  | Refined grain | >1 serving/day | 5 | 1 | 5 |
|  |  | 1 serving/day | 4 | 2 | 4 |
|  |  | 4-6 servings/week | 3 | 3 | 3 |
|  |  | 1-3 servings/week | 2 | 4 | 2 |
|  |  | Rarely or never | 1 | 5 | 1 |
|  | Salt-preserved vegetable | >1 serving/day | 5 | 1 | 5 |
|  |  | 1 serving/day | 4 | 2 | 4 |
|  |  | 4-6 servings/week | 3 | 3 | 3 |
|  |  | 1-3 servings/week | 2 | 4 | 2 |
|  |  | Rarely or never | 1 | 5 | 1 |
| Animal food | Milk | >1 serving/day | 1 | 1 | 1 |
|  |  | 1 serving/day | 2 | 2 | 2 |
|  |  | 4-6 servings/week | 3 | 3 | 3 |
|  |  | 1-3 servings/week | 4 | 4 | 4 |
|  |  | Rarely or never | 5 | 5 | 5 |
|  | Dairy products | >1 serving/day | 1 | 1 | 1 |
|  |  | 1 serving/day | 2 | 2 | 2 |
|  |  | 4-6 servings/week | 3 | 3 | 3 |
|  |  | 1-3 servings/week | 4 | 4 | 4 |
|  |  | Rarely or never | 5 | 5 | 5 |
|  | Fish and aquatic products | >1 serving/day | 1 | 1 | 1 |
|  |  | 1 serving/day | 2 | 2 | 2 |
|  |  | 4-6 servings/week | 3 | 3 | 3 |
|  |  | 1-3 servings/week | 4 | 4 | 4 |
|  |  | Rarely or never | 5 | 5 | 5 |
|  | Egg | >1 serving/day | 1 | 1 | 1 |
|  |  | 1 serving/day | 2 | 2 | 2 |
|  |  | 4-6 servings/week | 3 | 3 | 3 |
|  |  | 1-3 servings/week | 4 | 4 | 4 |
|  |  | Rarely or never | 5 | 5 | 5 |
|  | Meat | >1 serving/day | 1 | 1 | 1 |
|  |  | 1 serving/day | 2 | 2 | 2 |
|  |  | 4-6 servings/week | 3 | 3 | 3 |
|  |  | 1-3 servings/week | 4 | 4 | 4 |
|  |  | Rarely or never | 5 | 5 | 5 |
|  | Pluck | >1 serving/day | 1 | 1 | 1 |
|  |  | 1 serving/day | 2 | 2 | 2 |
|  |  | 4-6 servings/week | 3 | 3 | 3 |
|  |  | 1-3 servings/week | 4 | 4 | 4 |
|  |  | Rarely or never | 5 | 5 | 5 |

*Note:* For fruit and vegetables, average serving sizes per day were calculated firstly and then group into 5 categories after ranking from high to low and scored 5,4,3,2 and 1, respectively. The other 12 food groups were grouped into 5 groups according to the intake frequencies by questionnaires·

**Table S2.** **Model selection process of the trajectory subgroups.** First, we tested and determined the number of trajectories based on the log Bayes factor, i.e. 2log_e_(B_10_), calculated as 2×(BIC_complex_-BIC_simpler_). Meanwhile, the minimum sample size in each trajectory group should be over 5%. Second, we tried combinations of different trajectory shapes to determine the optimal shape of each trajectory based on the log Bayes factor as well. The table below presented the selection process.

| **Shapes of Subgroups** | | | | **BIC** | **Average posterior probability** | | | | | **Minimum sample size (%)** | **2log_e_(B_10_)*** | |  |
| --- | --- | --- | --- | --- | --- | --- | --- | --- | --- | --- | --- | --- | --- |
| **1. To determine the number of trajectories** | | | | | | | | | | | | |  |
| **Subgroup 1** | | **Subgroup 2** | |  | **Subgroup 1** | | **Subgroup 2** | | |  |  | |  |
| quadratic | | quadratic | | -132473 | 0.964 | | 0.899 | | | 20.56 |  | |  |
| **Subgroup 1** | **Subgroup 2** | | **Subgroup3** |  | **Subgroup 1** | **Subgroup 2** | | **Subgroup3** | |  |  | |  |
| quadratic | quadratic | | quadratic | -129011 | 0.908 | 0.899 | | 0.929 | | 5.23 | 3462 | |  |
| **2. To determine the shape of each trajectory** | | | | | | | | | | | | | |
| **Subgroup 1** | **Subgroup 2** | | **Subgroup3** |  | **Subgroup 1** | **Subgroup 2** | | | **Subgroup3** | **Minimum sample size (%)** | | **2log_e_(B_10_)** | |
| linear | linear | | linear | -129000 | 0.899 | 0.907 | | | 0.93 | 5.21 | | NULL | |
| linear | linear | | quadratic | -129005 | 0.907 | 0.899 | | | 0.931 | 5.21 | | -10 | |
| linear | linear | | cubic | -129009 | 0.907 | 0.899 | | | 0.93 | 5.23 | | -8 | |
| linear | quadratic | | linear | -129005 | 0.907 | 0.899 | | | 0.93 | 5.21 | | -10 | |
| linear | quadratic | | quadratic | -129009 | 0.907 | 0.899 | | | 0.931 | 5.21 | | -8 | |
| linear | quadratic | | cubic | -129012 | 0.898 | 0.908 | | | 0.93 | 5.21 | | -6 | |
| linear | cubic | | linear | -129009 | 0.907 | 0.899 | | | 0.929 | 5.23 | | -8 | |
| linear | cubic | | quadratic | -129014 | 0.907 | 0.899 | | | 0.929 | 5.24 | | -10 | |
| linear | cubic | | cubic | -129019 | 0.907 | 0.899 | | | 0.929 | 5.24 | | -10 | |
| quadratic | linear | | linear | -129002 | 0.908 | 0.899 | | | 0.929 | 5.21 | | -4 | |
| quadratic | linear | | quadratic | -129007 | 0.908 | 0.898 | | | 0.93 | 5.21 | | -10 | |
| quadratic | linear | | cubic | -129012 | 0.908 | 0.898 | | | 0.93 | 5.21 | | -10 | |
| quadratic | quadratic | | linear | -129006 | 0.908 | 0.899 | | | 0.928 | 5.23 | | -8 | |
| quadratic | quadratic | | quadratic | -129011 | 0.908 | 0.899 | | | 0.929 | 5.23 | | -10 | |
| quadratic | quadratic | | cubic | -129016 | 0.908 | 0.898 | | | 0.93 | 5.22 | | -10 | |
| quadratic | cubic | | linear | -129011 | 0.908 | 0.899 | | | 0.928 | 5.24 | | -10 | |
| quadratic | cubic | | quadratic | -129015 | 0.908 | 0.899 | | | 0.929 | 5.24 | | -8 | |
| quadratic | cubic | | cubic | -129018 | 0.909 | 0.899 | | | 0.927 | 5.01 | | -6 | |
| ***cubic*** | ***linear*** | | ***linear*** | ***-128970*** | ***0.908*** | ***0.898*** | | | ***0.93*** | ***5.19*** | | ***64*** | |
| cubic | linear | | quadratic | -128975 | 0.907 | 0.898 | | | 0.93 | 5.19 | | -10 | |
| cubic | linear | | cubic | -128979 | 0.908 | 0.898 | | | 0.93 | 5.19 | | -8 | |
| cubic | quadratic | | linear | -128974 | 0.907 | 0.898 | | | 0.931 | 5.19 | | -8 | |
| cubic | quadratic | | quadratic | -128979 | 0.908 | 0.898 | | | 0.93 | 5.19 | | -10 | |
| cubic | quadratic | | cubic | -128983 | 0.907 | 0.898 | | | 0.93 | 5.21 | | -8 | |
| cubic | cubic | | linear | -128973 | 0.908 | 0.898 | | | 0.93 | 5.22 | | 2 | |
| cubic | cubic | | quadratic | -128977 | 0.908 | 0.898 | | | 0.93 | 5.23 | | -8 | |
| cubic | cubic | | cubic | -128982 | 0.908 | 0.898 | | | 0.93 | 5.22 | | -10 | |

*Note:* log Bayes factor (2log_e_(B_10_)) ≈ 2×[Δ BIC] = 2×[BIC_complex_– BIC_simpler_]

**Table S3. Associations between quintiles of rhPDI and ruPDI and aging trajectories based on multinomial logistic regression model.**

| **Diet index** | **Median of  diet index** | **Medium-degree vs Slow aging** | | **High-degree vs Slow aging** | |
| --- | --- | --- | --- | --- | --- |
|  |  | N^*^ | OR (95% CI) | N^*^ | OR (95% CI) |
| **Quintile of rhPDI (N)** |  | | | |  |
| Q1 (1717) | 21 | 952 | Ref | 110 | Ref |
| Q2 (2222) | 24 | 1072 | 0.69 (0.60, 0.80) | 104 | 0.58 (0.42, 0.80) |
| Q3 (2720) | 25 | 1200 | 0.58 (0.50, 0.67) | 114 | 0.47 (0.35, 0.64) |
| Q4 (1191) | 27 | 518 | 0.58 (0.49, 0.69) | 60 | 0.54 (0.37, 0.78) |
| Q5 (2341) | 29 | 951 | 0.54 (0.47, 0.63) | 106 | 0.50 (0.36, 0.68) |
| **Quintile of ruPDI (N)** |  | | | |  |
| Q1 (2341) | 19 | 951 | Ref | 106 | Ref |
| Q2 (1191) | 21 | 518 | 1.07 (0.92, 1.26) | 60 | 1.08 (0.75, 1.55) |
| Q3 (2720) | 23 | 1200 | 1.07 (0.94, 1.21) | 114 | 0.95 (0.70, 1.28) |
| Q4 (2222) | 24 | 1072 | 1.28 (1.12, 1.47) | 104 | 1.17 (0.86, 1.59) |
| Q5 (1717) | 27 | 952 | 1.86 (1.60, 2.15) | 110 | 2.01 (1.46, 2.76) |
| *Notes:* Slow aging: Slow aging trajectory, Medium-degree: medium-degree accelerated aging trajectory, High-degree: high-degree accelerated aging trajectory. N^*^: Number of participants in medium-degree accelerated aging trajectory or high-degree accelerated aging trajectory. Model adjusted for age, gender, education level, marital status, smoking status, drinking status, chronic disease count and animal food index (as continuous). | | | | | |

**Table S4. Odds ratios and 95% CIs for aging trajectories, according to individual food groups and categories variables frequency**

| **Food** | **Frequency** | **Medium-degree vs Slow aging** | **High-degree vs Slow aging** |
| --- | --- | --- | --- |
|  |  | OR (95% CI) | OR (95% CI) |
| Whole grain | >1 servings /day | 0.91 (0.77, 1.07) | 0.72 (0.49, 1.08) |
|  | 1 servings /day | 0.82 (0.72, 0.93) | 0.69 (0.51, 0.94) |
|  | 4-6 servings /week | 0.66 (0.56, 0.77) | 0.68 (0.47, 0.98) |
|  | 1-3 servings /week | 0.69 (0.63, 0.75) | 0.64 (0.52, 0.79) |
|  | Rarely or never | Ref | Ref |
| Fresh fruit | ≥ 2 servings/day | 0.70 (0.60, 0.82) | 0.84 (0.59, 1.21) |
|  | 1.5-2 servings/day | 0.43 (0.37, 0.50) | 0.53 (0.38, 0.74) |
|  | 1-1.5 servings/day | 0.79 (0.69, 0.92) | 1.08 (0.78, 1.49) |
|  | 0.5-1 servings/day | 0.92(0.76, 1.11) | 1.20 (0.80, 1.80) |
|  | < 0.5 servings/day | Ref | Ref |
| Fresh vegetable | > 4 servings/day | 0.48 (0.42, 0.54) | 0.52 (0.39, 0.68) |
|  | 3-4 servings/day | 0.55 (0.47, 0.63) | 0.52 (0.38, 0.71) |
|  | 2.5-3 servings/day | 0.47 (0.41, 0.52) | 0.43 (0.34, 0.55) |
|  | 2-2.5 servings/day | 0.65 (0.57, 0.73) | 0.56 (0.44, 0.72) |
|  | < 2 servings/day | Ref | Ref |
| Rhizomes | >1 serving/day | 1.13 (0.70, 1.82) | 0.53 (0.13, 2.24) |
|  | 1 serving/day | 0.92 (0.73, 1.16) | 0.58 (0.31, 1.08) |
|  | 4-6 servings/week | 0.74 (0.63, 0.88) | 0.73 (0.49, 1.08) |
|  | 1-3 servings/week | 0.85 (0.78, 0.92) | 0.74 (0.61, 0.89) |
|  | Rarely or never | Ref | Ref |
| Legume | >1 serving/day | 0.56 (0.39, 0.80) | 0.76 (0.38, 1.53) |
|  | 1 serving/day | 0.84 (0.72, 0.97) | 0.85 (0.62, 1.17) |
|  | 4-6 servings/week | 0.75 (0.66, 0.85) | 0.59 (0.44, 0.80) |
|  | 1-3 servings/week | 0.85 (0.78, 0.92) | 0.68 (0.56, 0.82) |
|  | Rarely or never | Ref | Ref |
| Sugar | >1 serving/day | 2.87 (0.30, 27.64) | 8.63 (0.54, 138.75) |
|  | 1 serving/day | 1.24 (0.89, 1.73) | 0.43 (0.13, 1.37) |
|  | 4-6 servings/week | 0.80 (0.55, 1.17) | 0.88 (0.38, 2.04) |
|  | 1-3 servings/week | 0.74 (0.65, 0.84) | 0.69 (0.50, 0.94) |
|  | Rarely or never | Ref | Ref |
| Refined grain | >1 serving/day | 1.57 (1.25, 1.97) | 1.27 (0.79, 2.05) |
|  | 1 serving/day | 0.89 (0.78, 1.03) | 0.76 (0.55, 1.05) |
|  | 4-6 servings/week | 0.59 (0.51, 0.70) | 0.33 (0.21, 0.53) |
|  | 1-3 servings/week | 0.68 (0.62, 0.74) | 0.50 (0.40, 0.62) |
|  | Rarely or never | Ref | Ref |
| Salt-preserved vegetable | >1 serving/day | 0.61 (0.26, 1.46) | 1.53 (0.35, 6.75) |
|  | 1 serving/day | 1.61 (1.21, 2.16) | 2.80 (1.71, 4.60) |
|  | 4-6 servings/week | 1.55 (1.22, 1.98) | 2.14 (1.35, 3.39) |
|  | 1-3 servings/week | 1.09 (1.00, 1.19) | 1.23 (1.01, 1.49) |
|  | Rarely or never | Ref | Ref |
| Milk | >1 serving/day | 0.56 (0.41, 0.77) | 0.36 (0.14, 0.88) |
|  | 1 serving/day | 0.66 (0.59, 0.73) | 0.57 (0.44, 0.74) |
|  | 4-6 servings/week | 0.54 (0.46, 0.63) | 0.36 (0.23, 0.56) |
|  | 1-3 servings/week | 0.57 (0.51, 0.63) | 0.49 (0.38, 0.64) |
|  | Rarely or never | Ref | Ref |
| Dairy products | >1 serving/day | 1.02 (0.52, 2.04) | 3.33 (1.30, 8.55) |
|  | 1 serving/day | 1.33 (1.14, 1.56) | 1.35 (0.97, 1.88) |
|  | 4-6 servings/week | 1.32 (1.13, 1.54) | 1.22 (0.86, 1.72) |
|  | 1-3 servings/week | 1.26 (1.16, 1.36) | 0.98 (0.81, 1.19) |
|  | Rarely or never | Ref | Ref |
| Fish and aquatic products | >1 serving/day | 0.67 (0.47, 0.95) | 0.66 (0.28, 1.53) |
|  | 1 serving/day | 0.56 (0.48, 0.64) | 0.64 (0.46, 0.89) |
|  | 4-6 servings/week | 0.51 (0.45, 0.57) | 0.49 (0.37, 0.67) |
|  | 1-3 servings/week | 0.52 (0.48, 0.57) | 0.58 (0.48, 0.71) |
|  | Rarely or never | Ref | Ref |
| Egg | >1 serving/day | 1.29 (0.88, 1.89) | 1.44 (0.69, 2.98) |
|  | 1 serving/day | 1.14 (0.99, 1.31) | 1.14 (0.86, 1.51) |
|  | 4-6 servings/week | 0.93 (0.81, 1.05) | 0.79 (0.59, 1.05) |
|  | 1-3 servings/week | 0.86 (0.79, 0.95) | 0.67 (0.55, 0.82) |
|  | Rarely or never | Ref | Ref |
| Meat | >1 serving/day | 0.80 (0.62, 1.03) | 0.87 (0.51, 1.49) |
|  | 1 serving/day | 0.83 (0.73, 0.94) | 0.80 (0.61, 1.05) |
|  | 4-6 servings/week | 0.74 (0.66, 0.84) | 0.71 (0.54, 0.93) |
|  | 1-3 servings/week | 0.79 (0.71, 0.87) | 0.63 (0.51, 0.80) |
|  | Rarely or never | Ref | Ref |
| Pluck | >1 serving/day | 1.27 (0.18, 9.07) | NA |
|  | 1 serving/day | 1.77 (1.43, 2.18) | 2.15 (1.43, 3.24) |
|  | 4-6 servings/week | 1.83 (1.64, 2.05) | 2.02 (1.59, 2.56) |
|  | 1-3 servings/week | 1.14 (1.02, 1.26) | 1.61 (1.29, 2.02) |
|  | Rarely or never | Ref | Ref |

*Note:* Slow aging: Slow aging trajectory, Medium-degree: medium-degree accelerated aging trajectory, High-degree: high-degree accelerated aging trajectory. The Models were multivariable-adjusted for age and gender. Abbreviations: OR, Odds ratio; CI, confidence interval.

**Fig. S1 Timeline of data collection**


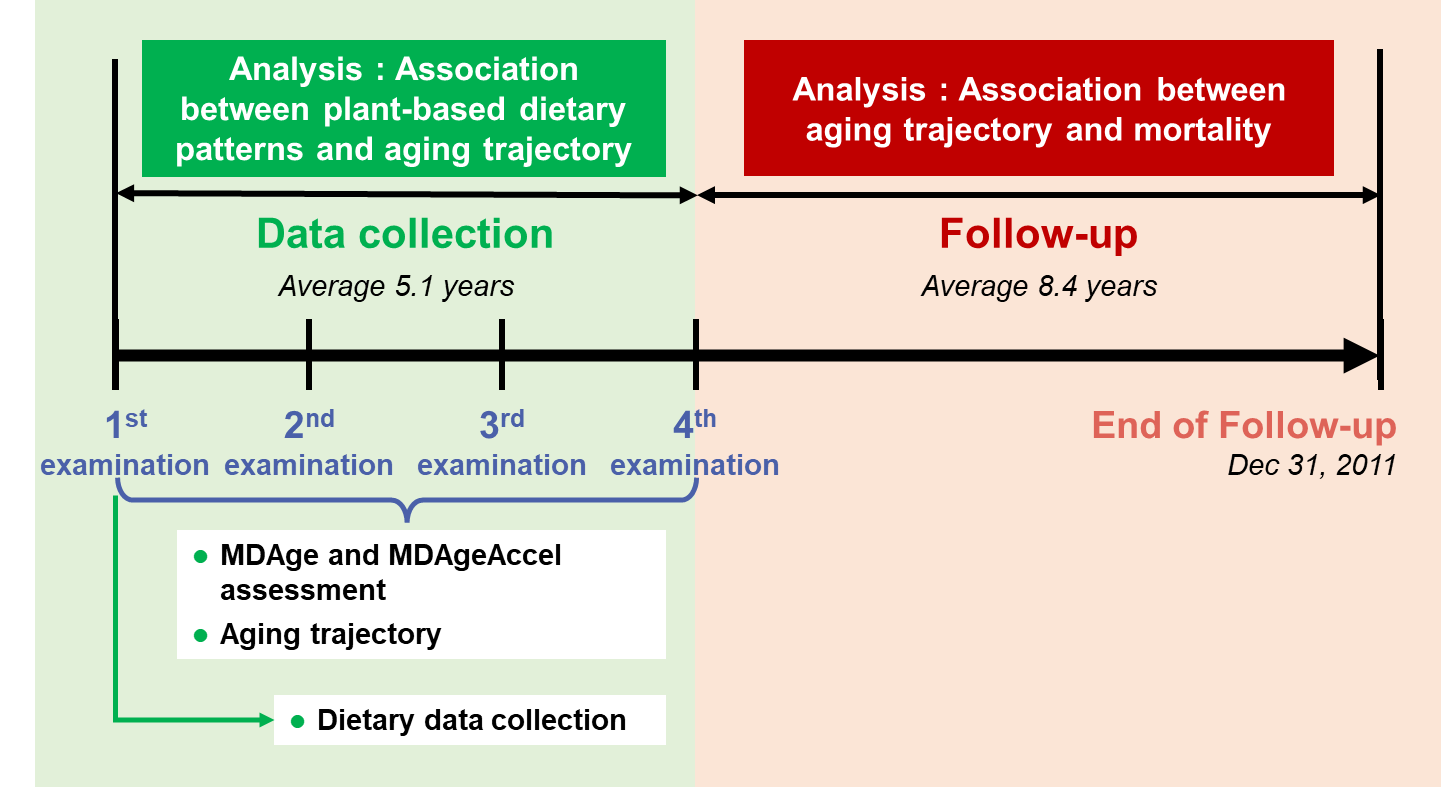


**Fig. S2. The pairwise Spearman correlation coefficients between different 14 food groups and 3 constructed food indices.**


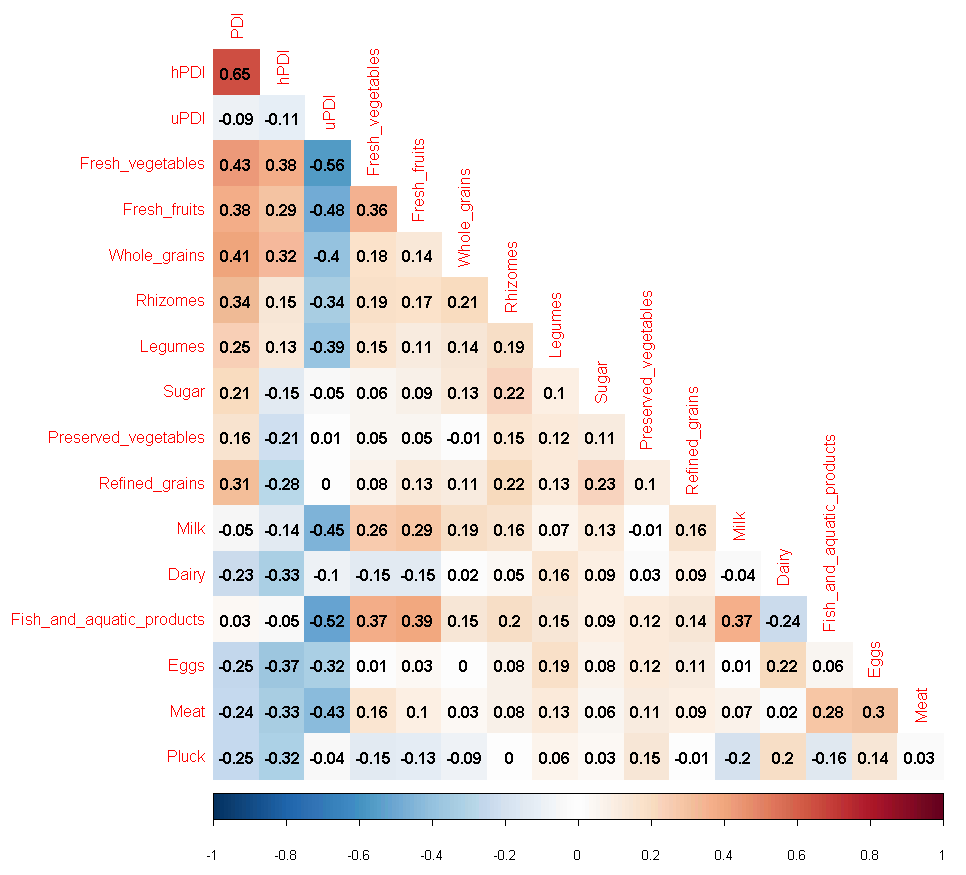


**Fig. S3. Aging trajectories across gender, education levels, married status, smoking status and drinking status.** (a) male, (b) female, (c) married, (d) unmarried, (e) middle school or below, (f) high school, (g) junior college, (h) college or above, (i) never smokers, (j) former or current smokers, (k) never drinkers, (l) former or current drinkers.


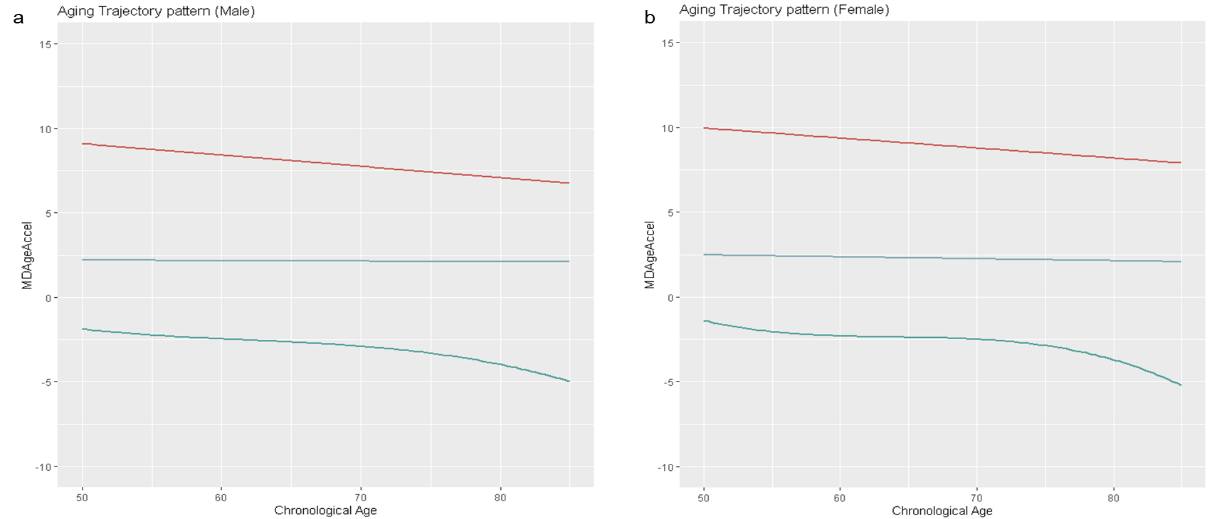

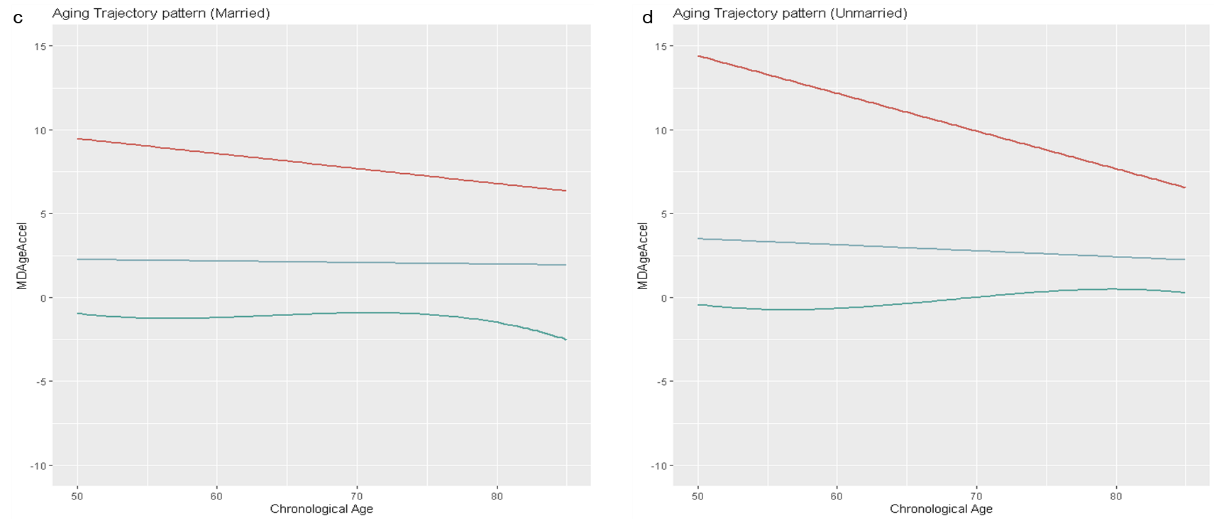


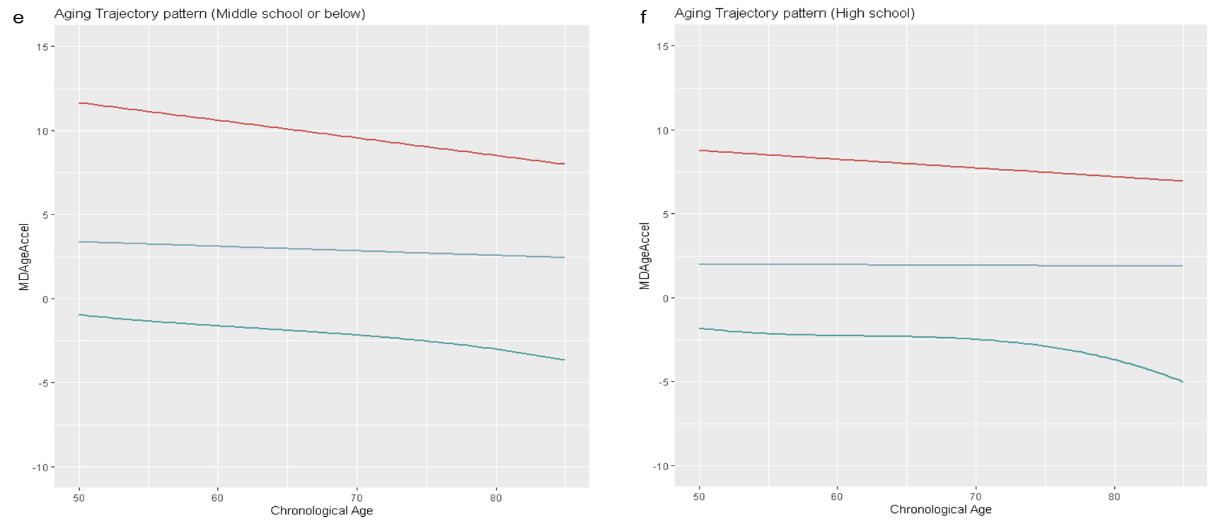

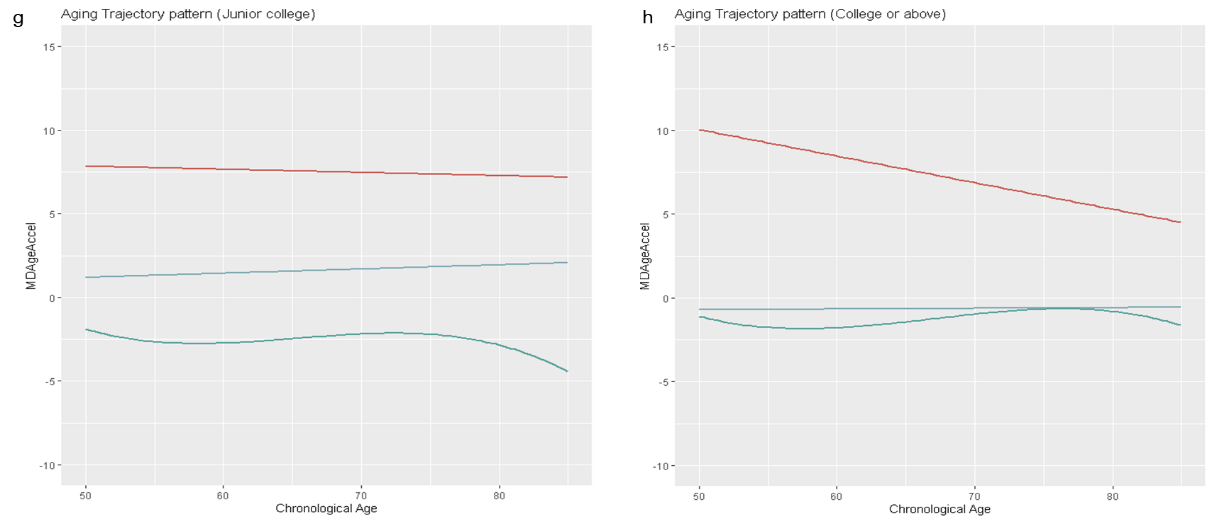

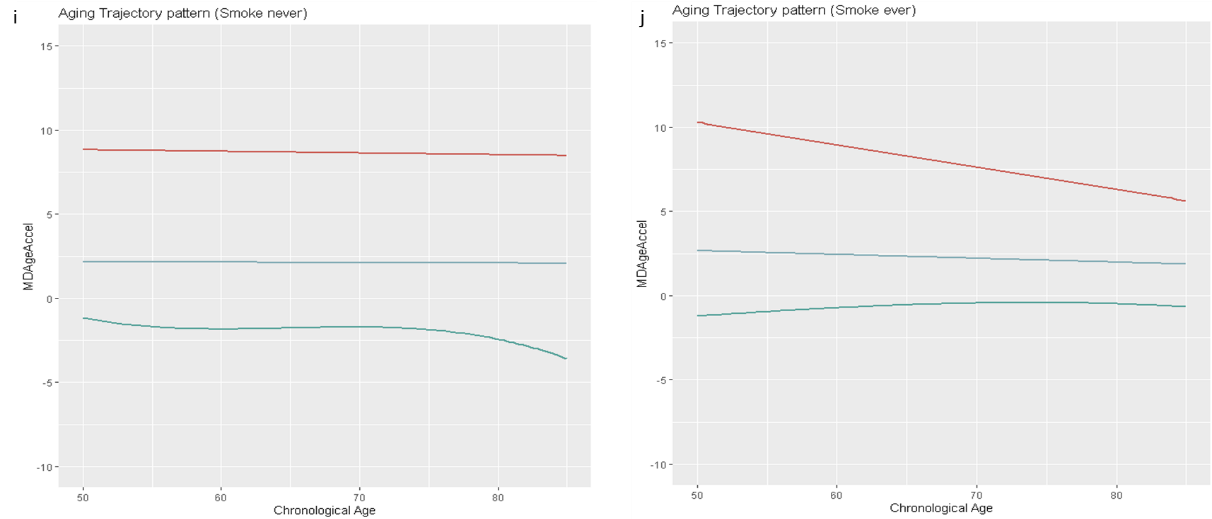


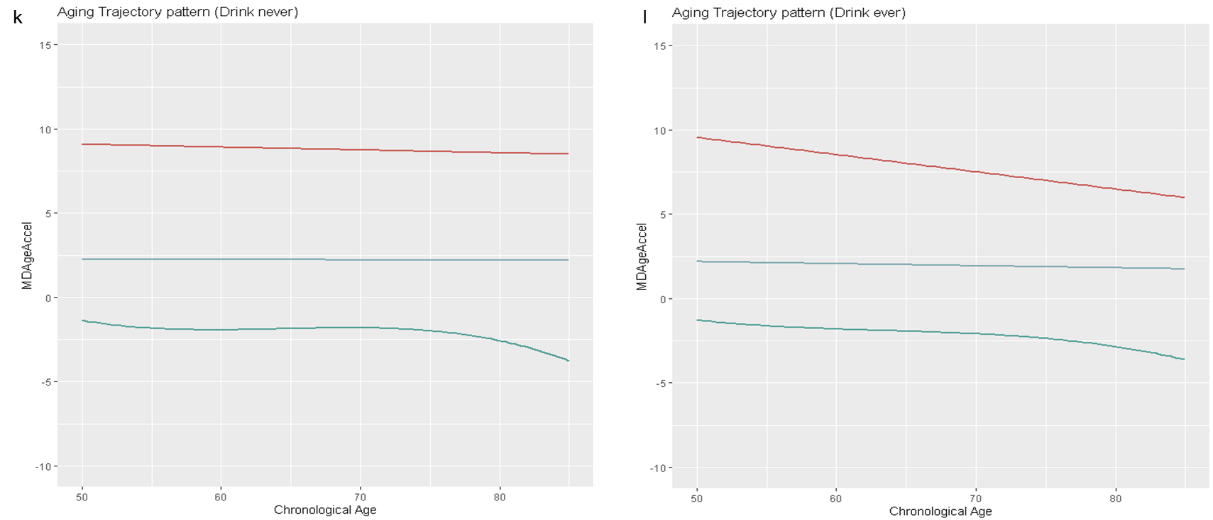


**Fig. S4. The pairwise Spearman correlation coefficients between different plant-based food groups and constructed food indices (PDI and rhPDI and ruPDI).**


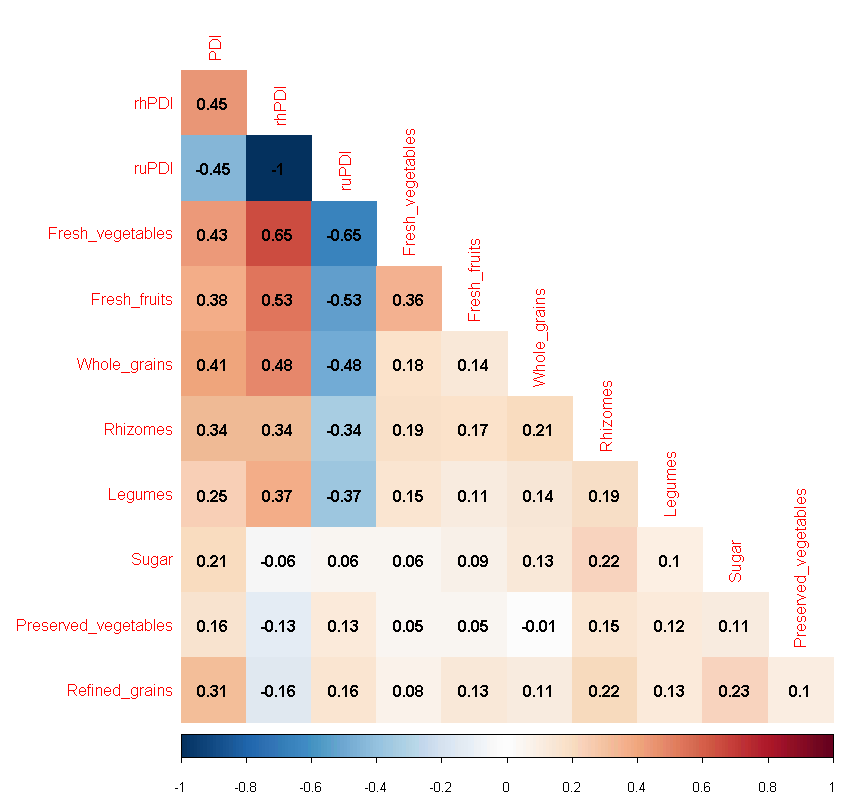

Supplement: Supplementary file 1 — Additional file 1: Table S1. Food groups and scoring. Table S2. Model selection criteria of trajectory subgroups. Table S3. Associations between quintiles of rhPDI and ruPDI and aging trajectories based on multinomial logistic regression model. Table S4. Odds ratios and 95% CIs for aging trajectories, according to individual food groups and categories variables frequency. Fig. S1. Timeline of data collection. Fig. S2. The pairwise Spearman correlation coefficient between different 14 food groups and 3 constructed food indices. Fig. S3. Aging trajectories across gender, education levels, married status, smoking status and drinking status. Fig. S4. The pairwise Spearman correlation coefficient between different plant-based food groups and constructed food indices (PDI and rhPDI and ruPDI). [file 12916_2023_2974_MOESM1_ESM.docx]
